# Becoming a mother when suffering from a chronic illness

**DOI:** 10.3389/fpsyt.2022.1059648

**Published:** 2023-01-23

**Authors:** Hélène Riazuelo

**Affiliations:** ^1^UFR Sciences Psychologiques et Sciences de l'Éducation (UFR SPSE), Université Paris Nanterre, Nanterre, France; ^2^Nephrology Psychosomatic Unit, Aura Paris Plaisance (APP), Paris, France

**Keywords:** pregnancy, perinatal care, links, chronic illness, psychotherapeutic treatment, reverie

## Abstract

**Introduction:**

The critical period of early motherhood when facing serious health problems constitutes a major public health issue. The disease may interfere with, influence, and compound the difficulties experienced over the course of pregnancy and during the parenthood processes. These processes are to be understood as a passage involving so many changes and fraught with difficulties leading to a series of psychological mobilizations. Illness also needs to be considered as a moment of transition, sometimes of severance, marking the lives of the people concerned in a more or less lasting way. Various developments are possible, some of which can be envisaged as leading to more positive outcomes, while others appear as if blocked or doomed to failure.

**Clinical data and method:**

This clinical study is the result of twelve analytically oriented psychotherapeutic follow-ups. The sessions took place weekly over periods ranging from 1 to 6 years. Some of the patients we met had become pregnant while they had a declared but not yet chronic nephropathy. The other patients were already on dialysis and had become mother before starting dialysis. There is also one instance of a pregnant patient on dialysis. In the background, there are also many women who talked about giving up fertility and motherhood. After an in-depth study of each follow-up, a cross-sectional study was conducted to identify the main themes.

**Results and discussion:**

Main considerations on the psychotherapeutic treatment: We regularly observe people who find it impossible to muster the internal resources that would enable them to deal with the trials they undergo in such situations. For the psychologist, there is a need to listen to archaic remnants. Gradually, in the space of psychotherapeutic work, possibilities of “reanimation” of the psyche emerge, an internal space that renews fantasmatic activity as it begins to be able to project into the external space and into the space of the sessions.

## 1. Introduction

Little has been written about the desire to become a parent and assuming all this entails when suffering from a serious somatic disease. In the medical field and also in psychology, work on women who are seriously ill and pregnant mainly relates to acute illnesses and cancer [for example (1–6)], or AIDS [for example (7–12)]. A growing interest in other severe conditions (13), including Chronic Kidney Disease (CKD), has however emerged in recent years (14–22).

This paradoxical, even violent interplay between life and death that a woman experiences in her body in a dramatically crosscutting fashion emerges from these writings. Tyer-Viola et al. (16) emphasize, along with others, that at the same time, there is an attempt to forget the illness by trying to experience a “normal” pregnancy like other women, with a parallel increase in anxiety when pregnancy may modify the symptoms of the illness. There is a clear need to manage potential complications during pregnancy, and it is essential to reflect on and conduct research into improving their management (20, 23). Robin summarizes matters in the psychoanalytical field when stating that during the course of the illness, the “threshold of permeability to the unconscious as also the preconscious is lowered; a lifting of repression takes place, which invasively gives way to regressive reminiscences and fantasies” [(24), p. 164].

Both periods are conducive to psychological reorganization during which the most archaic experiences are heavily drawn on. Thus, the psychological processes at work during pregnancy and becoming a mother intersect or intermingle with those set in motion after the announcement and treatment of a serious somatic illness. In this case, the maternal psyche is assailed by traumatic aspects. Anxiety and diffuse anguish are also perceptible. Close family and relatives are also seriously affected.

Pregnancy in women with chronic disease, and more particularly kidney failure, is still rare, especially when the condition deteriorates and the patients are on hemodialysis.^1^ The evolution of pregnancy can then become unfavorable due to the frequency of complications. The clinical and physiological disturbances observed an increase in the degree of kidney failure. With the improvement of hemodialysis techniques, the fertility of women suffering from chronic renal deficiency has improved considerably (particularly since the introduction of erythropoietin, which allows more women to have normal cycles). Although increasing successes are now being reported, the outcome of pregnancy for women undergoing dialysis remains extremely uncertain and these women have to deal with massive restrictions. “Intensification of hemodialysis, with up to five or six sessions a week (as opposed to the usual three weekly sessions), peritoneal dialysis, or even the temporary combination of these two methods, is essential for sufficient quality of the amniotic fluid” [(25) p. 121]. Any pregnancy in a woman with kidney disease should be considered as being at risk. During monitoring sessions, the full reality of bodily functions is omnipresent. Psychologically, it should also be borne in mind that end-stage renal disease involves a possibly traumatic confrontation with invasive techniques without which the patient can no longer survive. She cannot preserve herself (26) and each dialysis conjures up an end-of-life situation (27, 28). Confronted by all this, the patient's imaginary projections are crushed, subdued under an operative way of thinking, where discourse is usually merely factual and reverie is well-nigh impossible.

## 2. Materials and methods

The methodology adopted here is qualitative. The clinical work discussed here stems from analytically oriented psychotherapeutic follow-ups with a psychoanalytical orientation in medical services, both in obstetrics and gynecology (one case study), and nephrology (eleven studies). The sessions took place weekly for periods ranging from 1 to 6 years.^2^ The first above-mentioned case study took place with a pregnant woman who had been on dialysis for about a year. The follow-up continued a few months after the birth of the child. For the eleven other follow-ups, kidney disease was known and was not yet chronic before pregnancy but became so afterward. The start of dialysis (in-centre hemodialysis for each of them) began in the year following the birth of the child or a few years later (at most two). For four of these women, psychotherapeutic follow-up started before their pregnancy (and hence the start of dialysis) and for the other seven, it started when they were put on dialysis, that is after the birth of their child.

All this took place against a background of encounters with other women we remember, who were unable to have a child and were dealing with the work of renunciation. Issues related to femininity, maternity, and fertility arose very regularly during psychotherapeutic interviews. Clearly, the disease also heavily affected family members, especially the father of the child. However, the focus here is mainly on the women themselves, who also talked about their spouses, and it is their perspective and their own representations that will be discussed here. Participants' names were anonymized. Written informed consent was obtained from all the participants for the publication of any potentially identifiable data included in this article.

After an in-depth case study, a cross-sectional survey was conducted to identify the main lines of thought. These are presented below.

## 3. Clinical results

### 3.1. A matrix experienced as “unwelcoming”

Listening to pregnant women, it is common to hear them talk about what they feel within their bodies, describing how the baby moves and kicks out. Sensoriality is omnipresent. Pregnant women suffering from nephrological disorders who consulted soon confronted the author with descriptions of the inside, functional workings of their maternal bodies. This was accompanied by a plethora of anatomical detail on the disease's progression as it hollowed out an unwelcoming and deficient environment for the unborn child. Additional medical information painted an even bleaker picture when the dialysis went a little less well than usual, adversely affecting the quality of the amniotic fluid. They, thus, considered their bodies as places where “everything (was) bad,” “ruined bodies” that were no longer good for anything, entirely negative. An alternative interpretation was to split the body up into its good parts (uterus) and bad parts (the kidneys that failed to function and disrupt the rest, including the uterus).

As they prepared to bring their infant out into the world, they questioned their ability to be “good enough” mothers [in Winnicott's sense (29)] and were vulnerable to depression as they considered themselves to be “unwelcoming” to their child. For some, these two temporalities, pregnancy and illness, proved impossible to articulate and elaborate on, with the thought processes relating to one movement blocking off those on the other. They thought of themselves as a “bad” container, a “bad nurse” right from the time of pregnancy as their renal function deteriorated. The process of psychological integration of the unborn baby, which S. Missonnier calls “psychic nesting” (30), was undermined. Feeling physically deficient, their very ability to carry the child psychically became threatened (31). Their imaginings and their representations of the unborn child became extremely tentative and sketchy as they went through pregnancy not knowing whether it would be carried through to term, whether the baby might suffer fetal pain, or whether their illness would develop further.

### 3.2. A skin rendered transparent

Pregnancy entails a dimension of going to see, of taking a look inside (32), of showing this unborn child and at the same time of keeping it secret, of preserving it from view. Let us recall that for Winnicott in *Human Nature*, “true female genital functioning tends to be hidden, when it is not in fact secret (...) the fantasy plays with the concealment within oneself, the hidden and the secret.” [(33) p. 65]. Genital representations find their fullest experience in identification with the mother, who is capable of acting and conceiving a child. In young girls' games, we observe this same tendency where the dimension of secrecy is essential. “The game of *Can you keep a secret?* belongs typically to the female side of human nature, just as fighting and pushing things into holes belong to the male side. Unless a girl can keep a secret she cannot become pregnant. Unless a boy can fight or push a train through a tunnel he cannot deliberately impregnate” (1988, p. 65-66). A glimpse of the imaginative elaboration of their dominant bodily functioning can, thus, be seen in such games. That secrecy is an intimate space that one seeks to protect against the outside world but that also functions as a barrier to archaic and oedipal anxieties. Through, thus, defining the notion of secrecy, we also grasp the pleasure we can feel in keeping a secret and then sometimes sharing it. Working on this theme with pregnant women may help them develop a capacity to turn toward their internal mode.

This inward drive is further encouraged by the technological advances in gynecology and obstetrics (ultrasound, monitoring, etc.). During their interviews with the author, pregnant women with an underlying or declared CKD all described their bodies as being pried into under everyone's gaze. This increased scrutiny that is lavished on them and their baby reassured them in part, but it also had a price tag attached to it psychologically as they felt as though they were being made transparent, with their insides constantly being probed by machines and needles. They spoke of a body that was no longer merely attacked internally by the disease but also assailed at its very limits. This accumulation of micro-traumas compounded their fears daily. Patients in nephrology regularly evoked this theme, stressing how burdensome and painful this was at a time when they ought to be providing a containing envelope for their baby. This containing function was regularly put to the test. In order to cope with this, the work of primary erogenic masochism^3^ that intertwines the life drive and the death drive is essential. I shall give as an example the case of Léonie.

*We met Léonie for the first time when she was hospitalized for 3 weeks. She had to stay in bed throughout her pregnancy and even a little longer as she was to undergo another operation as soon as the baby was born. She was 5 months pregnant. She had kidney disease (due to diabetes) but also cardiac and digestive problems. She was connected to multiple catheters and an operation was scheduled after childbirth to restore her digestion. The seriousness of her medical complications weighed so heavily on the author that she felt trapped, unable to look beyond that immediate reality of her bedridden condition. Léonie explained that she cared about this baby above all else and that she had waited such a long time for it to come. She and her partner had wanted a child for several years now and the waiting became all the more painful as her additional somatic problems emerged, suddenly, on top of her already recognized heart problems. She was ready “to put up with anything, to endure everything, to wait week after week as long as (she could) keep (her) child.” To begin with, the couple's desire to have a child emerged slowly and quietly, with the idea of starting a family a little later when the time came... but she now saw a major difference, talking about a pregnancy and a child that were “vital” to her. It was “what gave (her) the strength to live,” as she put it. Having learned of her illness, she knew that it was going to be chronic for the rest of her life but she then wanted this child more than anything else to “prove to (herself), to show that (she could) also give life.” Her body “cannot let (her) down at all levels,” it must also be able to “give life.” Her comments as to her inner bodily workings were harsh and brutal, with only the womb being preserved as a bubble of life. She was a carrier of death, but she would also be a giver of life... She talked of this pregnancy, this child, as coming to repair her psychically as well as physically, her body in itself. Some days, she had real death anxieties, feeling the peripheral parts of her body become numb (peripheral neuropathy being a common symptom in the later stages of diabetes). Even though she had not yet reached that condition, she was acutely aware of such complications and even evoked the sensation of herself gradually being transformed into a corpse. As the unborn infant moved inside her, knocking, this brought her out of these moments of morbid stupor. She also looked forward to examinations of the baby, where the gynecologists would tell her that he was doing well. Admittedly, he was rather small and a Cesarean section was already being envisaged before the end of pregnancy, but he was developing quite normally. Léonie kept repeating the words “completely normal” to herself. Even dependent as she was on medicine and treatment, she was going to give birth to a child in good health. She was able to carry him and ensure that he developed normally. Despite moments of doubt and worry, she felt like a good enough mother (according to Winnicott's meaning)*.

Inevitably, the scopic impulse is of course exacerbated by the reinforced medical monitoring needed to ensure that pregnancy proceeds well. Each medical indicator is watched assiduously. This is psychologically demanding but also reassures the mother-to-be when the baby's and her own medical results are good. Technology and medication are indispensable to maintaining life.

### 3.3. The dream baby^4^

This hollow female organ, the uterus, is at the center of these women's concerns and calls upon them in particular at the phantasmic level. In reference to Klein, the mother's body is fantasized as a recipient of combined wealth, namely including the father's penis and new babies to come. The infant in the fantasy is an internal object among others such as the breast, penis, feces, and child, all related to the receptacle containing all, the “good” as well as the “bad,” that is to say, the maternal interior. This recalls the box that contains all secrets, as M. Cournut-Janin has already pointed out: “Between Dora's jewelry box and the secret that Winnicott's little girl knows how to keep, the metaphor of a box and what it conceals is constructed: the female body, which is rich in ‘transposable' content (penis, feces, child, jewelry, buttons, not forgetting the blood, which is promising, of the menstrual period)” [(38), p. 57]. The interior of the female body can also be full of other types of objects, which can be subsumed under the category of waste. At a fantastical level, the treasured baby or the jewel baby is evoked, but the womb may also be seen as a receptacle for non-productive discharge or even poison. In women with CKD, these phantasmic babies are present as in many women, but the feeling is all the more acute and the divisiveness all the more pronounced.

The baby as treasure is there to fill out, restore, and enhance child-like desires. In nephrology, this is especially true, as the pregnant patients feel a sense of fulfillment, a kind of narcissistic elation: being pregnant for them is a true victory. The infant is a treasure, a movement of life. Even as the disease emerges or asserts itself, it becomes eclipsed by the marvelous mundanity of the life of a woman who goes on her way just like any other, meeting a man and then becoming pregnant. These women spoke of real “healing.” For women with kidney disease, the baby also comes in to mend them, to show them that their bodies are not just sick and fantasized as “bad” or “damaged” but able to bring forth life. They, thus, prove to be fertile when few thought it was possible. This baby that normalizes her life can become a saving grace (and hence is largely idealized), an ornament to the mother-to-be, while the baby too can be adorned like their mother, reinforcing their femininity phantasmically.

However, the fetus is also potentially dangerous to the mother and can encroach on or invade her maternal body. For women with CKD, pregnancy can actually degrade their kidney functions. The baby can, thus, be associated with deteriorating maternal health generating a form of ambivalence that can sometimes take on a persecutory aspect.

These women tend to think that their inside is not being appropriately fitted to receive the unborn child in optimal conditions. The liquid environment is imperfectly purified, not adequately renewed, thus reactivating fantasies of a soiled, damaged, cloacal matrix. The anal dimension is largely present. The baby can then be fantasized as being attacked by this “waste” or even being part of it. The idea of a form of “invasion” by the disease is regularly mentioned.

### 3.4. The “motherhood constellation”^5^

Again referring to the approach adopted by Melanie Klein, the primordial anguish of the girl, the woman, concerning her inner body relates to the fear of seeing herself ravished, damaged within her body, above all through her genital organs. The mother's body is the place where all that is desirable is located, the place for investigations of origins and the very mainspring of life. As a result, the child, especially the daughter, is full of hatred toward her mother, whom she wishes to attack in order to steal what she hides inside her body. This is followed by anguish linked to the fear of reprisals and of seeing the inside of her body stolen away and destroyed (40). Her own ability to bear children is destined to play a similar role, to prove to her the unaltered nature of her inner self, for which healthy, vigorous offspring will provide proof. Conversely, any flaw in the child's successful development will revive the mother's anxieties relating to her invisible, unverifiable, and worrying inner body and the ensuing reprisals she fears from her mother. It is also the fear of having one's internal or intimate treasures (penis, feces, or fetus) and the gem baby taken away, especially by the mother with whom the daughter had tried to rival and who may now want to take back what belongs only to her, especially the father's child. Thus, some women suffering from kidney failure spoke about their fear of a broken filiation and how they felt it was for them impossible to become a mother. Many childhood desires were re-awakened, and especially their past “cravings” for the maternal body. Their difficulties surrounding their own fertility and pregnancy came as an echo, a form of punishment for their infantile sexual desires.

Confrontation with their own mothers for these women can be painful. Ambivalence and aggression are regularly palpable and may be accompanied by a feeling of guilt. This is exacerbated when the kidney disease is hereditary.

Moreover, when faced with this debt of life (41–43) toward their mother who brought them into the world, the infant may seem to provide a way to settle matters. However, the question remains as to whether the patient will be able to carry things through to childbirth, after which there may still remain a complex debt that cannot be removed. We need to remember that these women talk of the life they are bearing within them but also of their possible early death. Well cared for, that outcome may still be remote, but the disease remains naggingly present, accompanied by the fear of their imminent demise. These women readily resort to mentioning their mother (sometimes a sister) as the person who will take care of their child if 1 day they are no longer able to do so. They also look to the coming months when they will have to organize their lives as mothers and fit all this in with their dialysis, already seeing themselves as mothers in control but only tenuously as they rely on treatment several times a week before having to recover. Their mother's presence by their side provides reassurance but also holds them within a form of dependence. They are to become mothers but regularly remain as if maintained in an infantile position, being dependent on the medical team and the machine itself in order to survive and give birth:

*Katia is the mother of a 2-year-old girl. She says she is lucky because she was able to have her child before dialysis. She is severely diabetic and pregnancy did not help but at least she has her child by her side. She hears her laughing, she plays with her and she talks about her “ray of sunshine.” She doesn't think about the future, about her failing eyesight and the treatment that is “eating out her life.” All she thinks about is her daughter. After a pause in the interview, she explains that the thrice weekly trips to undergo dialysis are becoming more and more painful for her as she leaves her daughter behind. She knows her child is in good hands with her mother, but the whole thing is becoming more and more unbearable. The situation is not just a “separation, like any woman going to work, but something else mixed in.” Katia feels unable to care for her daughter, unable to run toward her or carry her anymore as she devotes attention to her arm where the fistula that allows her to be connected to the dialysis machine is located. She feels she is failing when off work for health reasons. Sometimes she is so tired that she finally entrusts her daughter to her mother so that she can sleep and recover. She is a mother but only “part-time.” She gets depressed. She wanted so much to be a mother and in the end her own mother takes more care of her daughter than she does. She feels guilty about her rivalry and aggressiveness toward her mother, which depresses her even more. Neither does she live up to her mother's standards. She talks about her childhood and what her mother did with her and for her, which she is not sure she can do with her daughter. She finds ways around the difficulties. Some days she feels creative enough and other days she thinks that she will never be able to give her daughter what she has received. She talks about a half-hearted transmission*.

## 4. Discussion: Clinical treatment shrouded in uncertainty

The major challenge within our health institutions is to sustain multi-professionalism in hospitals in order to understand and cater to the patient as a whole. Due to cross-analysis conducted by different professionals, care is thought out and shared by several people with different skills. A kind of “constellation” of health and care professionals is essential here.

The recent pandemic has stimulated new thinking on health issues and innovative concepts such as “one health” are emerging. A recent French ministerial report published in February 2022 promotes this philosophy to reflect on the “lessons of the crisis.” The World Health Organization defines health as “a state of complete physical, mental and social wellbeing and not merely the absence of disease or infirmity”^6^ The patient's health is, thus, considered in all its complexity, in relation to those close to her and the world around her. From this perspective, psychologists have a duty to be attentive to the analysis of institutional dynamics. We actively participate in the reflections of the medical and care teams at various institutional levels involving the hospitalization service staff at multidisciplinary meetings (doctors, nurses, social workers, dieticians) during which the teams formulate new demands in our direction. These meetings are also an opportunity to discuss ongoing follow-up. Interstitial times are also important, with the psychologist meeting caregivers and physicians. The psychologists then devote time to passing on information to the medical and care teams.

As psychologists and psychotherapists working in these medical services, we are confronted with a “clinic of the real” (44), where the experiences of trauma, mourning, and loss intersect. In these psychosomatic services, the harsh realities of clinical work can severely affect us. Faced with the burden of bodily representations, we try to think with the patient, in the dynamics of exchanges, of the place the spoken word can occupy, enabling the emergence of representations of a body closer to the fantasy, drawing away from the clutches of immediacy. In the medical world, the real daily challenge is to maintain the stability of the psychological framework (in the broadest sense of the term) offered to our patients, considering the sick person in her somatic and psychic wholeness, in connection with her entourage and environment. To avoid becoming overwhelmed by the grim realities of the sickness, we need as psychologists, to find our own temporality and retain our own capacity for reverie (in the sense of Bion) with regard to our patients.

Clearly, patients suffering from chronic diseases where their very survival is at stake experience considerable distress. The process of dialysis itself is a potent reminder of mortality and proximity to death becomes deafening as first object relations move into the foreground, shrouded in uncertainty. Psychic disorganization can be massive. We regularly observe patients who are distraught and unable to find the internal resources to cope with the experience. Here, the psychologist has a duty to listen to archaic traces as the body, in dialysis, appears in all its reality and vulnerability. As practitioners, we regularly have to listen to speech devoid of prosody, lacking modulation and affects, and having to remain attentive to the unspeakable. Gradually, in the space of psychotherapeutic work, possibilities for the “reanimation” of the psyche emerge, as part of an internal space that takes up a phantasmic activity as it begins to be able to project into the external space and the space of the sessions.

Psychological or psychotherapeutic follow-up provides a reflective appraisal so as gradually to recover the pleasure of thinking and allow for the reintegration of possible traumas. During the patient's discourse, time regularly freezes over. It remains somehow suspended, sometimes in a lasting way. Faced with such discontinuity, the patient needs to find regularity in the relationship and consistency in the medical and nursing teams, as well as in our own psychotherapeutic framework. This therapeutic structure needs constancy for the patient to emerge from the situation of dependence and reappropriate experience as a “continuity of being” (as understood by Winnicott).

The arrival of the baby is marked by serious medical constraints and by the limitations imposed by the disease. In the course of psychotherapeutic follow-up, we gradually try to leave more room for reverie. The patient can then take the risk of investing more calmly in the baby, her baby, as pregnancy progresses and she feels it move within her. The challenge is to differentiate and specify the sensations specific to her pregnant body and the fetus and, thus, rely on them so that the representations surrounding and enveloping this unborn baby can develop and become richer and greater in number.

These women may sometimes wish to commence psychotherapeutic work to begin the process of mourning for their fertility and the child. The work of renunciation is accomplished gradually and transmission will take place elsewhere, moving toward other objects and other projects.

## 5. Conclusion

When the child is born and terminal kidney disease occurs, it may be important to “de-crystallize” this period as the different psychic movements may possibly collide and become paralyzed. Pregnancy, the birth of the child, and sometimes the child itself are all part of the history of the illness. Demands on the child, its life debt, can be embroiled in what is happening with the illness. The child is caught up in the same loop as the machine (the artificial kidney that drains the patient three times a week), making them live like the machine.

However, even when carried along with the illness, pregnancy remains a moment of life where the work of creation, both physiological and psychological, is at work. In particular, it affords an opportunity to pass on the message that beyond the disease, life goes on.

A natural follow-up to this work would be the study of early interactions between mothers on dialysis or about to be on dialysis and their children. This is a surprisingly understudied area that would be important to develop.

## Data availability statement

The original contributions presented in the study are included in the article/supplementary material, further inquiries can be directed to the corresponding author.

## Ethics statement

Ethical review and approval was not required for the study on human participants in accordance with the local legislation and institutional requirements. The patients/participants provided their written informed consent to participate in this study. Written informed consent was obtained from all the participants for the publication of any potentially identifiable data included in this article.

## Author contributions

The author confirms being the sole contributor of this work and has approved it for publication.

## Footnotes

^1^Hemodialysis usually involves the patient being dialyzed three times a week, for four-hour sessions. They are then connected to an artificial kidney that cleans the blood week after week. Kidney transplantation provides an alternative in some cases.

^2^These follow-ups are currently completed and were collected over a period of about 10 years during the author's practice as a clinical psychologist.

^3^Which we can define as the first psychic connection realizing an entanglement between the life drive and the death drive necessary for psychic life. This makes it possible to withstand the tensions of excitation that are inevitable and necessary for psychic life [according to Rosenberg (12)].

^4^Aulagnier (34); Lebovici (35); Soulé (31); Cupa-Pérard (36); Nerson-Sachs (37).

^5^Stern (39).

^6^ https://solidarites-sante.gouv.fr/IMG/pdf/contribution_conseil_scientifique_8_fevrier_2022_one_health.pdf
